# Value-Based Care and Accountable Care Organizations: Implications for Early Autism Diagnosis and Access to Quality Care

**DOI:** 10.3390/bs15101354

**Published:** 2025-10-04

**Authors:** Kyle M. Frost, Heather E. Hsu, Marisa Petruccelli, Rebecca McNally Keehn, Hanna Rue, Angela Beeler, Sarabeth Broder-Fingert

**Affiliations:** 1 Department of Pediatrics, University of Massachusetts Chan Medical School, Worcester, MA 01655, USA; marisa.petruccelli@umassmed.edu (M.P.); angela.beeler@umassmemorial.org (A.B.); 2 Division of Pediatric Health Services Research, Department of Pediatrics, Boston Medical Center, Boston, MA 02118, USA; heather.hsu@bmc.org; 3 Department of Pediatrics, Indiana University School of Medicine, Indianapolis, IN 46202, USA; mcnallyr@iu.edu; 4 LEARN Behavioral, Baltimore, MD 21209, USA; hanna.rue@learnbehavioral.com

**Keywords:** autism, value-based care, health services, payment reform, accountable care organizations

## Abstract

The incentives in fee-for-service healthcare payment systems to increase clinical volume often work in opposition to efforts to coordinate care or improve care delivery in partnership with community-based services. There has been increasing interest in and adoption of value-based care as an alternative healthcare delivery model in which clinician reimbursement is based on measures of healthcare quality and patient outcomes, meant to shift the focus from generating volume toward providing more efficient, coordinated care. In this commentary, we discuss potential benefits, challenges, and unintended consequences of this fundamental shift in payment systems and the specific implications for autism services, highlighting critical areas of focus for future research and policy development.

## 1. Introduction

Autism is a lifelong neurodevelopmental disability which is often identified in the toddler years based on differences in social communication and the presence of restricted and repetitive behaviors. Recent estimates indicate that 1 in 31 children in the United States are autistic (Shaw et al., 2025). From birth to age 5 years, the standard of care is “individualized, developmentally-appropriate, and intensive” intervention to support communication, cognitive development, and adaptive functioning (Hyman et al., 2020, p. 20). As children grow from toddlerhood to school-age, services often transition to emphasize academic and social skills and school participation. As youth reach transition-age to adulthood, services become scarce and are more likely to emphasize occupational and housing support and co-occurring mental health or medical conditions. Throughout the lifespan, the presence of co-occurring physical and mental health conditions is the norm rather than the exception. Support needs for autistic people vary widely, with some benefiting from intensive, wraparound care and others requiring relatively little specialized support. Even within individuals, support needs can vary substantially across the lifespan.

Autism services are unique in that they are often intensive and address behavioral and physical health needs, requiring engagement and collaboration across various service systems. The spread of services across professions (e.g., neurology, psychiatry, psychology, speech-language therapy, behavior analysis) and service systems (e.g., medical, educational) often results in fragmentation, making service navigation a full-time job for many caregivers (Brewer, 2018). Health system infrastructure, including constraints on reimbursement, limited clinician time, and complex payor requirements, further contributes to poor continuity and coordination of care (Walsh et al., 2020).

Consistent with the Quadruple Aim of healthcare, autistic people and their families deserve coordinated, efficient, high-quality care that is integrated across medical and behavioral health and supports wellbeing at the population level (Bodenheimer & Sinsky, 2014). Unfortunately, structural features of the United States healthcare system, including payment models, have contributed to challenges in achieving this goal. The goal of this commentary is to consider how payment reform might shape healthcare access and quality for autistic youth.

## 2. Value-Based Care

Many patients, clinicians, and policy experts have noted flaws in the predominant fee-for-service healthcare payment systems (Ho & Sandy, 2014). These payment models, in which health systems are reimbursed a negotiated fee for each service provided, often either explicitly or implicitly encourage volume over value and often fail to incentivize quality of care, cost containment, coordination of care, and connection to community-based services (Wynne, 2016). Value-based care is an alternative model in which clinician reimbursement is based on measures of healthcare quality and patient outcomes (NEJM Catalyst, 2017). Accountable care organizations (ACOs), which aim to improve quality and reduce cost through population health management and capitated payments, are a widespread approach to value-based reform in the United States (Muhlestein et al., 2022). In ACO care models, groups of clinicians or hospital systems receive a set budget for a defined population of patients (generally within a geographic region) and a defined period of time, which often includes incentives for achievement of quality benchmarks, care coordination, and connection to community-based organizations. This shift to a capitated payment system is intended to encourage more efficient, coordinated care, rather than incentivizing volume (as with fee-for-service).

Payment system and policy reform may be a powerful tool to move towards achieving the Quadruple Aim of healthcare by improving access to high-quality, coordinated, and efficient care for autistic people and their families and supporting a resilient clinical workforce. However, the success of value-based payment systems in improving care quality and reducing healthcare spending has been variable, with aspects of program design strongly influencing its realized benefits (Pandey et al., 2023). We argue that, due to the unique features of the autism service delivery system, ACOs should explicitly consider impacts to autism services in the design and implementation of payment reform and be wary of potential unintended consequences to service quality and access (Table 1). Given the current service landscape and unique needs of the autism population, there are several key considerations needed to advance ACO implementation in ways that benefit autistic people and their families.

## 3. Timely Access to Early Diagnosis and Intervention

Access to high-quality early intervention is associated with reduced need for long-term educational services and a significant cost-offset within a few years (Cidav et al., 2017; Dimian et al., 2021). In order for families to access autism-specific services, particularly applied behavior analysis (ABA), payors require a medical diagnosis of autism. Traditionally, diagnosis is determined through standardized evaluation conducted by specialty clinicians, leading to a substantial bottleneck in early service access, with too few clinicians (with limited geographic reach) trained in autism diagnosis (McNally Keehn et al., 2024). For children under three who are eligible for Early Intervention, autism services are largely funded by a combination of federal, state general and earmarked funds, and the healthcare system (primarily by Medicaid; Infant and Toddler Coordinators Association, 2023). The cost of providing early diagnostic and intensive services is substantial—up to $80,000 annually—but this early investment may yield significant long-term savings of up to $20,000 annually over 18 years for the education system (Cidav et al., 2017). Once children reach school age and receive services through their public school system, a subset of intervention costs shift to the education sector, while others remain covered by medical insurance or are paid for out-of-pocket.

A challenge in implementing ACOs for the general pediatric population is the limited opportunity for short-term healthcare cost savings, with most savings taking the form of long-term return on investment (Brykman et al., 2021). Unfortunately, ACOs’ imperative to reduce costs in the short term may create incentives to delay the autism diagnostic process. Although earlier diagnosis and timely access to high-quality intervention can lead to better outcomes and lower lifetime service costs across both the healthcare and education sectors, from the ACO’s perspective, care of autistic children under three is a money-losing proposition, with much of the cost-savings reaped by the education system or social service agencies in the long term or by another health system when the individual no longer belongs to that ACO.

In sum, the current ACO model does not reward the system which performs early screening, diagnosis, and treatment for improving long-term outcomes. Yet, by carefully considering how to integrate autism care into the ACO model, there is an opportunity to develop value-based metrics that promote best practices. This can be achieved by explicitly incentivizing early screening, appropriate referral to diagnostic services, and enrollment in early intervention. Furthermore, the more flexible payment structure could be leveraged to support more timely access to diagnosis by supporting non-billable activities that increase efficiency. For example, this could include triaging patients to providers with the necessary training/skill level, supporting shared-care models among primary and specialty care, and allowing for increased teaming and interdisciplinary collaboration.

## 4. Coordination of Care Across Service Systems

A diagnosis of autism is often rendered by a licensed professional in a medical or behavioral healthcare setting. However, post-diagnostic services are provided by community service agencies or in educational settings. American Academy of Child and Adolescent Psychiatry guidelines recommend use of multiple treatment modalities, including behavioral, communication, and educational interventions (Volkmar et al., 2014). Indeed, many families report accessing behavioral, speech-language, and occupational therapy services, with behavioral services accessed at a higher intensity than other service modalities (Monz et al., 2019).

For children with early developmental delays, state early intervention systems that provide services from birth to 3 years are often a family’s first touch point for care, even prior to receiving a formal diagnosis. When children turn 3, those eligible for special education may receive services through the educational system, while those who do not qualify for special education rely on healthcare services solely through their medical or behavioral health plan benefits. Many children access a combination of medical, behavioral health, rehabilitation, and educational services, posing a unique challenge to care coordination.

All 50 states have now mandated ABA coverage for autism through private insurance and Medicaid, leading to rapid expansion in the availability of this service (Barry et al., 2017) and dramatic growth in the number of behavior analysts (Behavior Analyst Certification Board, 2025; McBain et al., 2020). Alongside this rapid expansion, challenges have emerged from the perspectives of payors and clinicians implementing ABA services within the medical model. ABA services are distinct from other insurance-based behavioral health services in that they are often delivered intensively, requiring significant financial, time, and personnel investment. For young children, particularly those under the age of six, clinical recommendations often include 20+ hours per week of one-to-one therapy (Council of Autism Service Providers [CASP], 2024). Although other behaviorally oriented professionals (e.g., psychologists) can provide and be reimbursed for ABA services in some states (depending on State regulations and payor requirements), Board Certified Behavior Analysts (BCBAs) have specific didactic and practical training in this intensive model of care. In many ABA specialty agencies, BCBAs design individualized intervention programs and supervise behavior technicians who implement them at a high level of intensity. Yet, behavior analysts are rarely trained to operate within a traditional medical model of care, in which payors have specific requirements around documentation of medical necessity, session notes, treatment plans, and progress monitoring that impact access to care (Kornack et al., 2014; Papatola & Lustig, 2016).

A goal of ACOs is to provide as much care as possible within the ACO network in order to reduce out-of-network costs and support care quality and coordination. “Leakage” is when a patient covered by the ACO obtains care from a non-ACO entity, which is undesirable from the perspective of the ACO (Zheng et al., 2018). Leakage is more common for pediatric autism services than for other chronic conditions, primarily driven by outpatient behavioral health services (e.g., ABA) provided in home- and community-based settings (Robinson et al., 2020). Most ACOs spend significant time and effort developing strategies to avoid leakage (Fisher et al., 2006) with the goal of improving quality, access, and care coordination while reducing cost (Gardner, 2016). However, in the case of autism services, leakage may be unavoidable, with many necessary (and, sometimes, intensive) services (e.g., ABA and specialty rehabilitation services) unavailable within the ACO, requiring families to seek support outside of the network. There is often poor coordination of care across medical, educational, and rehabilitation services, since different service systems may have varying expectations or support for collaboration (Rizk et al., 2023). Moreover, loopback to the medical home is also challenging, and often it falls on caregivers to assume the role of case manager.

ACO implementation is often associated with dedicated care coordination services (Anderson & Chen, 2019). In developing a model for autism services, this opportunity for increased care coordination may support service access and reduce negative outcomes on caregivers filling this role. In addition, it may be necessary for ACOs to define networks more broadly by formalizing relationships with home- and community-based services in order to bring behavioral health and rehabilitation services in network (Robinson et al., 2020). This has the potential to facilitate more coordinated care across service systems and avoid disincentivizing access to a variety of necessary services.

## 5. Measurement of Quality of Care for Autism-Specific Services

The diversity of support needs that characterizes the autism spectrum and variability in presence of co-occurring conditions across individuals and over time poses challenges to the measurement of intervention progress and quality of care (Joseph et al., 2024). Unlike most other health conditions, autism is a lifelong neurodevelopmental disability for which symptom reduction is often not an appropriate or desired goal for intervention. Instead, intervention goals are often tied to improving adaptive skills and supporting educational or occupational goals or reducing the negative impact of co-occurring behavioral health concerns or maladaptive behavior. Appropriate and feasible intervention goals must be tailored to the individual and family. The wide array of intervention targets and approaches makes developing generalized outcome assessments or benchmarks challenging.

A key component of value-based care includes measurement of outcomes to evaluate quality of care, which is tied to payment incentives (Squitieri et al., 2017). In the case of autism services, it is unclear what an appropriate outcome assessment or service quality metric would be. An International Consortium for Health Outcomes Measurement (ICHOM) working group recently published an autism-specific standardized outcome set which highlights the breadth of relevant outcome domains (e.g., social communication, anxiety, sleep, family functioning) and the wide range of measures required to assess such disparate outcomes (Joseph et al., 2024). At present, payors often mandate specific norm-referenced measures to justify service intensity and monitor treatment outcomes for ongoing service authorization (e.g., Vineland Adaptive Behavior Scales, 3rd edition (Vineland-3; Sparrow et al., 2016)). Many commonly used instruments, including the Vineland-3, have inadequate psychometric properties for evaluating change over time, making them a poor fit for monitoring progress toward goals (Frazier et al., 2025b; Joseph et al., 2024). Such norm-referenced measures often do not meaningfully inform treatment goals or quantify progress and add burden on clinicians and families who complete them (Frazier et al., 2025b). Because many intensive service providers (e.g., behavior analysts) are not trained in administering these required norm-referenced measures, families are often required to seek out specialists for re-evaluation, adding to the access bottleneck.

As payment systems shift, there is a unique opportunity to develop meaningful autism-specific care quality metrics that meet payor requirements. Such metrics should be developed collaboratively among experts in autism services and psychometrics, autistic people and their families, and payors. Standardized metrics of interest might focus on child and family wellbeing and engagement rather than child outcomes that are inherently variable and difficult to interpret when aggregated (Bethell et al., 2025). Process-based metrics, that evaluate clinician’s application of evidence-based clinical decision making or measurement-based care, may also be valuable (Schwartzman et al., 2023). Given the diversity of treatment approaches, service setting, and child characteristics across studies, it is unsurprising that research examining the effects of intervention intensity, duration, and modality have been mixed (e.g., Frazier et al., 2025a; Linstead et al., 2017; Sandbank et al., 2021; Virués-Ortega, 2010; Yoder et al., 2020). As such, it is critical that decisions around optimal service type, intensity, and duration are individualized to child and family needs and determined collaboratively between families and their clinical team. An ongoing challenge for the field will be to establish data-driven guidelines for optimal dose and type of services, taking into account specific child and family characteristics and preferences.

## 6. Influence of Autism on Broader Healthcare Access and Utilization

Autistic children and their families report a high level of unmet healthcare needs and a lower quality of care than for other disabilities (Drahota et al., 2020; Kogan et al., 2008; Menezes et al., 2021). They have difficulty accessing primary and specialty services, as evidenced by higher rates of emergency department utilization among autistic youth (Hunt et al., 2018; Iannuzzi et al., 2022) and lower rates of preventive and well-child visits (DeGuzman et al., 2022). Unmet healthcare needs are associated with higher rates of emergency department utilization, while having a medical home is associated with lower rates of emergency department utilization, suggesting that coordinated care has a meaningful impact on service utilization for this population.

While children with autism may be primed to gain from care coordination and behavioral health management imperatives integrated into many ACO contracts, an ACO’s ability to invest care coordination and behavioral health resources tailored to the autism population depends on whether the higher cost of care for children with autism is budgeted for. ACOs use the concept of “risk adjustment” to set quality benchmarks/performance metrics and payments based on the medical or social complexity of their patients (Cholera et al., 2023). At the population level, performance metrics may evaluate (and therefore incentivize) such benchmarks as percentage of children receiving yearly well-child visits, immunization status, percentage of enrollees screened for social service needs, percentage of children receiving preventive dental services, and hospital re-admission rate. For example, it is well established that children with autism face significant challenges in obtaining dental care and are more likely to delay or forgo oral health evaluations. Longstanding misconceptions surrounding autism and childhood immunizations have made vaccine hesitancy prevalent in this population. Therefore, it is almost guaranteed that the autism population will be less likely to meet such metrics.

Although ACO payment models commonly allocate more money for medically complex patients (e.g., those with chronic illnesses or social barriers to care), there is little evidence for whether they adequately account for complexity related to neurodevelopmental disabilities like autism. Payment and quality assessment models that fail to account for medical complexity may underestimate costs of care and unfairly penalize clinicians caring for children with autism or caring disproportionately for autistic children with higher support needs. Unchecked, this incentivizes healthcare agencies to “cherry pick” patients who are more likely to meet performance metrics, creating the potential for health disparities in which those with the greatest need for care are least likely to access it (Alexander, 2020; Savva et al., 2023). On the other hand, adequate risk-adjustment paired with thoughtful performance metrics have the potential to allay these concerns; for example, a performance metric to conduct developmental screeners and complete appropriate follow-up and referral would support access to care for children with autism and other developmental delays. We suggest that ACOs devote considerable attention to understanding and meeting quality metrics that support service access for the autism population.

## 7. Conclusions

Structures of healthcare systems, including payment systems, have substantial power to shape healthcare access and outcomes to improve population health and achieve the Quadruple Aim for autistic people. At the same time, systems that do not account for the needs of specific populations run the risk of excluding them from their benefits or causing substantial harm through exclusion and low-quality care, creating or exacerbating health inequities. The goal of this commentary was to consider value-based care in general, and ACOs specifically, with regard to areas of promise and potential pitfalls for supporting healthcare for autistic people (Table 1). Rather than advocate for any optimal payor system, our goal was to outline considerations for high-quality care and the ways in which ACOs may or may not improve upon quality of care in autism services.

Regardless of the payor system, we believe that high-quality care that is coordinated across service systems and clinicians is necessary to improve the health and wellbeing of autistic people. Even within fee-for-service systems that continue to dominate healthcare, the addition of flexible funds to cover non-billable services, like care coordination and family navigation, has the potential to improve quality of care while reducing service duplication and gaps in service access. Furthermore, the development of reliable and meaningful measures of patient-centered outcomes that are important to autistic people and their families is necessary to support the implementation of quality metrics to incentivize high-quality care. No matter the type of payment reform, it is imperative that policy decision making includes people who understand autism services and how they relate to the broader health services landscape in order to avoid undermining service access.

## Figures and Tables

**Table 1:** Potential areas of promise and pitfalls in the implementation of autism-specific healthcare services in accountable care organizations.

Challenge	Promise	Pitfall
1. Timely access to early diagnosis and intervention	Development of value-based metrics that promote early screening, diagnosis, and enrollment in early intervention	Limited opportunity for cost savings that may disincentivize early diagnosis and intervention access
2. Coordination of care across service systems	Expansion of resources for care coordination and broadly defined networks that facilitate coordinated access to medical, behavioral health, and rehabilitation services	Challenges accessing home- and community-based services unavailable within the ACO’s network
3. Measurement of quality of care for autism-specific services	Opportunity to define patient- and family-centered outcomes focused on wellbeing and service engagement	Use of inappropriate norm-referenced measures to justify service intensity that may limit access to care
4. Influence of autism on broader healthcare access and utilization	Reimbursement that accounts for the higher cost of care associated with autism coupled with performance metrics that incentivize service access	Inadvertent penalties for clinicians who deliver services to autistic children, particularly with higher support needs

## Data Availability

Not applicable.

## Abbreviations

The following abbreviations are used in this manuscript:

ACO	Accountable Care Organization
ABA	Applied Behavior Analysis
